# Down-Regulation of miRNA-708 Promotes Aberrant Calcium Signaling by Targeting Neuronatin in a Mouse Model of Angelman Syndrome

**DOI:** 10.3389/fnmol.2019.00035

**Published:** 2019-02-13

**Authors:** Naman Vatsa, Vipendra Kumar, Brijesh Kumar Singh, Shashi Shekhar Kumar, Ankit Sharma, Nihar Ranjan Jana

**Affiliations:** ^1^Cellular and Molecular Neuroscience Laboratory, National Brain Research Centre, Manesar, India; ^2^School of Bioscience, Indian Institute of Technology, Kharagpur, India

**Keywords:** Angelman syndrome, UBE3A, miR-708, neuronatin, parvalbumin, calcium signaling

## Abstract

The expression of ubiquitin ligase *UBE3A* is paternally imprinted in neurons and loss of function of maternally inherited *UBE3A* causes Angelman syndrome (AS), a neurodevelopmental disorder characterized by severe intellectual disability and motor disturbances. Over activation of UBE3A is also linked with autism. Mice deficient for maternal Ube3a (AS mice) exhibit various behavioral features of AS including cognitive and motor deficits although the underlying molecular mechanism is poorly understood. Here, we investigated possible involvement of miRNA in AS pathogenesis and identified miR-708 as one of the down-regulated miRNA in the brain of AS mice. This miR-708 targets endoplasmic reticulum resident protein neuronatin (a developmentally regulated protein in the brain) leading to decrease in intracellular Ca^2+^. Suppression of miR-708 or ectopic expression of neuronatin increased the level of intracellular Ca^2+^ and phosphorylation of CaMKIIα at Thr286. Neuronatin level was significantly increased in various brain regions of AS mice during embryonic and early postnatal days as well as in parvalbumin-positive GABAergic neurons during adulthood with respect to age-matched wild type controls. Differentiated cultured primary cortical neurons obtained from AS mice brain also exhibited higher expression of neuronatin, increased intracellular basal Ca^2+^ along with augmented phosphorylation of CaMKIIα at Thr286. These results indicate that miR-708/neuronatin mediated aberrant calcium signaling might be implicated in AS pathogenesis.

## Introduction

UBE3A belongs to HECT (homologous to E6-AP C-terminus) domain family of E3 ubiquitin ligase, which plays an essential role in selective targeting of proteins for ubiquitination and degradation (Huibregtse et al., 1995). In addition to its ubiquitin ligase activity, UBE3A also have transcriptional co-regulator function (Ramamoorthy and Nawaz, 2008). The *UBE3A* gene exhibits paternal-specific imprinting in the neuronal tissue and the loss of function of maternally inherited *UBE3A* results in Angelman syndrome (AS), a neurodevelopmental disorder typically characterized by severe delay in developmental milestones, intellectual disability, lack of speech and epilepsy along with several other accompanying features particularly excessive laughter and sleep disturbances (Albrecht et al., 1997; Kishino et al., 1997; Matsuura et al., 1997; Fang et al., 1999; Yamasaki et al., 2003; Williams et al., 2010). Although, majority of AS cases are caused by the deletion of maternal chromosome 15q11-q13 (where multiple genes reside), identification of point mutations in *UBE3A* gene in subset of AS strongly implicated *UBE3A* as the candidate gene for AS. Interestingly, duplication, triplication or gain-of-function mutations in *UBE3A* gene are also linked with autism suggesting that the expression and activity of UBE3A must be precisely regulated during brain development (Glessner et al., 2009; Yi et al., 2015; Xu et al., 2018). The *Ube3a*-maternal deficient mouse exhibits many of the behavioral phenotype observed in AS and thus serves as a typical model system to understand disease pathogenesis (Jiang et al., 1998). These mice display not only the deficit in cognitive and motor functioning, but also audiogenic seizure, anxiety-like behavior and disturbance in circadian clock and sleep homeostasis (Jiang et al., 1998; Heck et al., 2008; Mulherkar and Jana, 2010; Godavarthi et al., 2012; Shi et al., 2015). Extensive investigation in this AS mouse model has revealed impairment in hippocampal long-term potentiation (LTP) along with abnormal activity in calcium/calmodulin dependent protein kinase-IIα (CaMKIIα), disrupted activity-dependent synaptic plasticity and imbalance in excitatory/inhibitory circuitry (Jiang et al., 1998; Weeber et al., 2003; Yashiro et al., 2009; Sato and Stryker, 2010; Wallace et al., 2012). These results strongly indicate that Ube3a plays a crucial role in regulating synaptic function. So far, several targets of Ube3a have been identified and many of them are found to be connected with synaptic function and plasticity (Greer et al., 2010; Sun et al., 2010).

MicroRNAs (miRNAs) are small non-coding class of endogenous RNAs (18–23 nucleotides in length), which are known to fine tune the gene expression by suppressing mRNA translation or its degradation (Bartel, 2009). Since its discovery, miRNAs have been demonstrated to be an integral part of the post-transcriptional regulatory machinery. Each miRNA could target multiple genes and is therefore expected to be a robust regulator of complex gene networks (Baek et al., 2008). In fact, they have been shown to play a significant role in regulating cell growth, differentiation, and development (Alvarez-Garcia and Miska, 2005; Fineberg et al., 2009; Inui et al., 2010). Large body of evidence also indicates crucial involvement of miRNAs in regulating neurogenesis, neuronal maturation, and synaptic plasticity (Coolen and Bally-Cuif, 2009; Fineberg et al., 2009; Im and Kenny, 2012). Hence, it is not surprising that altered expression of miRNAs could be linked to the defect in neurogenesis, synaptic function and plasticity associated with many neurological disorders. Indeed, deregulated miRNAs expression are increasingly implicated in the pathogenesis of various psychiatric and neurodevelopmental disorders (Li et al., 2008; Beveridge and Cairns, 2012; Mellios and Sur, 2012; Mundalil Vasu et al., 2014; Liu et al., 2015; Hicks and Middleton, 2016; Wu et al., 2016; Thomas et al., 2017). But, so far no miRNA has been reported to be regulated by UBE3A or deregulated in AS, which led us to investigate possible alteration in expression of miRNAs and their link with disease pathogenesis using AS mouse model.

Here we report miR-708 as one of the dysregulated miRNA in the AS mice brain. We subsequently characterized neuronatin (Nnat) to be one of the major targets of miR-708. Neuronatin is a small membrane protein localized predominantly in the endoplasmic reticulum (ER) and exists in two isoforms (α and β having 81 and 54 amino acids respectively). It is predominantly expressed in the developing brain and thought to play an important role in the brain development (Joseph et al., 1995; Dou and Joseph, 1996; Joseph, 2014). In the adult brain, Nnat expression is mostly restricted to the structure associated with the limbic system (Vrang et al., 2010). Although the precise role of Nnat in brain is not clearly understood, it has been shown to trigger neural induction in embryonic stem cells through increasing intracellular Ca^2+^ by antagonizing sarco/endoplasmic reticulum Ca^2+^ -ATPase (SERCA) and its abnormal function is associated with certain types of epilepsy (Lin et al., 2010; Sharma et al., 2011, 2013). We demonstrate that miR-708 regulates intracellular Ca^2+^ homeostasis by targeting Nnat thus causing aberrant Ca^2+^ signaling in AS mice brain.

## Materials and Methods

### Materials

Dulbecco’s modified Eagle’s medium (DMEM), all reagents for SDS-PAGE and mouse monoclonal antibodies against β-actin (A5316) and parvalbumin (PV, P3088) were purchased from Sigma. Fetal bovine serum, antibiotics and all primary culture reagents were purchased from GIBCO. Lipofectamine^®^ 2000, optiMEM, trizol reagent, Alexa fluor (AF)-594 and 488 conjugated secondary antibodies, mouse monoclonal V5 antibody (R960-25) were purchased from Invitrogen. Rabbit polyclonal anti-Ube3a (SC-25505), anti-pThr286 CaMKIIα (SC-12886) were purchased from Santa Cruz Biotechnology. Rabbit polyclonal and mouse monoclonal anti-Nnat antibody (ab27266 and ab181353) and mouse monoclonal anti-CaMKIIα antibody (611292) were from Abcam and BD Bioscience respectively. Mouse specific Nnat siRNA oligonucleotides (SC-149937, a pool of three target specific 20–30 nucleotide siRNA) and control siRNA (SC-37007, scrambled sequences) were also purchased from Santa Cruz Biotechnology. HRP-conjugated anti-rabbit and anti-mouse IgG were obtained from Vector laboratories. The 3′-UTR of *Nnat* was amplified (using following primers: Forward 5′-ttatcgtcgaccccagctcccagccct-3′ and Reverse 5′-atatgcggccgctttttggtgcacccccact-3′) and cloned in psiCHECK-2 vector (Promega). The 3′-UTR was cloned between the *Xho*I and *Not*I restriction site of the vector. The construction of Nnatα and Nnatβ plasmids (in pcDNA with V5 tag) has been described earlier (Sharma et al., 2011).

### Animal Experimentation and Ethics Statement

Ube3a heterozygous mice (129-Ube3atm1Alb/J) were obtained from Jackson Laboratory and maintained in animal house facility of the Institute. Animals were kept on a 12 h light/dark cycles, with food pellets and water *ad libitum*. Female *Ube3a*-maternal deficient mice (*Ube3a*m-/p+) were bred with male wild type mice (*Ube3a*m+/p+) to generate AS mice and subsequently confirmed by genotyping as described earlier (Jiang et al., 1998). Mice belonging to postnatal age groups P1, P5, P10, and P60 were used for experiments. In primary neuronal culture experiment, pregnant dams at embryonic day 16 (E16) was used. All animal experiments were conducted in accordance with the approval of the Institutional Animal Ethics Committee of National Brain Research Centre. Mice were handled strictly according to guidelines defined by the Committee for the Purpose of Control and Supervision of Experiments on Animals (CPCSEA), Ministry of Environment and Forestry, Government of India.

### Neuronal Cell Culture, Transfection, and Luciferase Assays

Mouse neuro 2a and HT22 cells (generously provided by Dr. Dave Schubert of Salk Institute, United States) were cultured in DMEM supplemented with 10% heat-inactivated fetal bovine serum and antibiotics penicillin/streptomycin. For regular transfection experiments, cells were plated onto six-well tissue culture plates at sub-confluent density. After 24 h of plating, cells were transfected with miR-708 mimic (5 nM) or inhibitor (50 nM) along with respective negative controls (10 picomoles each/well) using Lipofactamine^®^-2000 according to the manufacturer’s instruction (transfection efficiency was about 50–60%) and 24 h after transfection, cells were processed for total RNA extraction or immunoblot analysis. In some experiment, *Nnat* 3′-UTR luciferase reporter vector (1 μg/well of 6-well tissue culture plate) was transfected into neuro 2a cells along with miR-708 mimic and 24 h of post-transfection, cells were harvested and subjected to dual luciferase assay according to the manufacturer’s protocol. *Nnat* expression plasmids (3 μg each/well of 6-well plate) were transfected into HT22 cells and 24 h later cells were processed for immunoblot analysis.

### Mouse Primary Neuronal Culture

Primary cortical neuronal cultures were prepared from E16 mouse embryo obtained from time-pregnant AS mice. Some part of the brain was used for genotype analysis. The cortex was isolated and trypsinized in Hank’s balanced salt solution containing sodium pyruvate (0.11 mg/ml), 0.1% glucose, 10 mM HEPES (pH 7.3), 0.25% trypsin and 1.2 unit/ml DNase at 37°C. Dissociated cells were plated on cover slips (about 200–300 cells/mm^2^) coated with poly-L-lysine in MEM Eagle’s media supplemented with 10% heat inactivated fetal bovine serum, 0.45% glucose, 1 mM sodium pyruvate, glutamax and penicillin/streptomycin. Fifteen hours of post-plating, whole media was replaced with neurobasal media supplemented with B27 and 2 mM glutamax along with penicillin/streptomycin. Half of the media was replaced every 3^rd^ day, and the culture was maintained for 14 days followed by subsequent experimental procedures.

### Immunoblotting Experiment

Mice were sacrificed by cervical dislocation, cortex from both the hemisphere was dissected out, immediately snaps frozen in liquid nitrogen and stored at -80°C. Collected brain tissues were homogenized in the ice cold RIPA lysis buffer (10 mM Tris, pH 7.4, 150 mM NaCl, 10 mM EDTA, 2.5 mM EGTA, 1% Triton X-100, 0.1% SDS, 1% sodium deoxycholate, 10 mM NaF, 5 mM Na_4_P_2_O_7_, 0.1 mM Na_2_VO_5_, complete protease inhibitor cocktail), lysates were sonicated briefly and centrifuged for 15 min at 15000 ×*g* at 4°C. Supernatants were collected, protein concentrations were determined by BCA methods and stored at -80°C in different aliquots for further use. For immunoblot analysis, samples were boiled with SDS-PAGE sample buffer for 5 min and equal amounts of proteins were resolved through SDS-PAGE followed by semidry transfer into nitrocellulose membrane and probing the blot with different antibodies as described earlier (Sharma et al., 2011). The primary antibodies and their dilutions used in this study were as follows: Nnat (1:3000), Ube3a (1:1000), pThr286CaMKIIα (1:3000), total CaMKIIα (1:3000), β-actin (1:5000), and V5 (1:3000).

### Immunofluorescence Staining

Mice were anesthetized with ketamine (100 mg/kg body weight) and xylazine (10 mg/kg body weight) and perfused transcardially with phosphate-buffered saline (PBS) followed by 4% paraformaldehyde (w/v) in PBS. Collected brains were subsequently kept in 4% paraformaldehyde for 24 h and then treated with 10, 20, and 30% sucrose (in PBS) followed by sectioning in freezing microtome (20 μm thickness). Serial brain sections were stored in PBS with 0.02% sodium azide at 4°C. For immunofluorescence staining, sections were first exposed to antigen unmasking reagent at 70°C for 45 min in a water bath and then washed several times with PBS, blocked with 2% BSA along with normal goat serum for 2 h and then incubated overnight with various primary antibodies (Nnat at 1:300 and PV at 1:500 dilutions). After several washings with PBS, sections were incubated with secondary antibodies conjugated either with AF-594 or AF-488 (used at 1:1000 dilutions) for 1 h, washed and then sections were counterstained with DAPI and observed using a fluorescence microscope (Apotome, Zeiss). For immunofluorescence staining of primary cortical neurons, cells grown on coverslips were washed with PBS, fixed with 4% paraformaldehyde in PBS for 20 min, permeabilized with 0.3% Triton X-100 in PBS for 5 min, washed 4–5 times, and then blocked and incubated with primary antibodies as above. Ube3a and pCaMKIIα Thr286 antibodies were used at 1:500 dilutions. After multiple washings with PBS, cells were incubated with appropriate fluorescent-labeled secondary antibody for 1 h, washed several times, mounted and observed under confocal microscope (Zeiss).

### miRNA Array Analysis and Quantitative RT-PCR

RNA was extracted from mouse cortical samples and neuro 2a cells using TRIzol reagent. The miRNA array analysis was performed by iLife Discoveries using Affymatrix miRNA 3 array. Data were first normalized using Affymatrix Expression Console followed by analysis using GeneSpring GX12.5 software to identify differentially expressed miRNA. Briefly, raw data sets were extracted from raw intensity file (CEL files) after scanning of slides. All the original microarray data (CEL files) for the control and test experiment was pre-processed using RMA (Robust Multichip Average) algorithm that consists of three steps: a background adjustment, Quantiles normalization and finally summarization. All above procedures were done by selecting default RMA algorithm using data adjustment and background correction in Affymetrix Expression Console 1.2.1.20. The normalized intensity files exported from Expression Consoles tool to GeneSpring GX 12.5 software for the differential miRNA expression analysis and fold change analysis.

For qRT-PCR analysis, cDNA was synthesized from total RNA, as per the procedures supplied with the reagent. qPCR for Nnat transcript was performed using power SYBR green PCR master mix (Applied Biosystems) on ViiA7 real time PCR system (ABI). The data was subsequently analyzed and expressed as fold change. RT-PCR product for Nnat was normalized against 18S RNA. Primer sequences for Nnat and 18S RNA were as follows: Nnat forward, 5′-GCTCATCATCGGCTGGTACA-3′; Nnat reverse, 5′-CTGGTCGAGAAGCACAGGAG-3′; 18S forward, 5′-GAGGGA GCCTGAGAAACGG-3′; 18S reverse, 5′-GTCGGGAGTGGGTAATTTGC-3′. PCR program used with the following condition: initial denaturation at 95°C/5 min followed by 40 cycles of denaturation at 95°C/15 s, annealing at 60°C/1 min, extension at 72°C/1 min. For qPCR of miR-708, cDNA was synthesized from total RNA using miScript II RT kit (Qiagen, Germany) followed by PCR using miScript SYBER Green PCR kit (Qiagen, Germany) as per manufacturer’s instructions on ViiA7 real time PCR system. Specific primers for miR-708 and RNU6 were purchased from Qiagen. The PCR program used for miRNA analysis was as follows: initial denaturation at 95°C for 5 min followed by 35 cycles of denaturation at 94°C/30 s, annealing at 55°C/30 s, extension at 72°C/30 s.

### Measurement of the Intracellular Ca^2+^

For intracellular Ca^2+^ measurement, cells were incubated with 5 μM Fluo-4 AM in HEPES buffer (130 mM NaCl, 5.4 mM KCl, 0.08 mM MgCl_2_, 1.8 mM CaCl_2_, 15 mM glucose and 20 mM HEPES, pH 7.4) for 30 min at 37°C. Subsequently cells were washed thrice with HEPES buffer and kept in the dark for 30 min to allow for complete dye de-esterification. Chamber slides containing fluo-4 AM loaded cells were mounted on the stage of an inverted laser scanning confocal microscope (Nikon) for live cell imaging. Time measurement studies for intracellular Ca^2+^ was performed using a 10x objective with 1.5x optical zoom. Cells were excited with the argon laser at 488 nm and the emitted fluorescence was measured using the 526 nm band pass filter. The images were acquired with a 12-bit peltier-cooled CCD camera. Baseline fluorescence intensity of fluo-4 was recorded for initial 100 s, after which thapsigargin (100 μM) was added to the cell and the recordings were performed continuously for maximum of 1000 s. For evaluating basal intracellular Ca^2+^, fluorescence intensity was recorded up to 300 s. The data acquisition and analysis were performed using the NIS elements AR software (Nikon). Fields containing 50–60 cells were randomly selected in measuring fluorescence intensity of individual cell. Each experiment was repeated thrice.

### Statistical Analysis

All experimental data were analyzed using Microsoft Excel software. Values were represented as mean ± SD. Student’s *t*-test was used to compared different groups of data and *P* < 0.05 was considered statistically significant.

## Results

### miR-708 Is Down-Regulated in AS Mice Brain

**FIGURE 1:**
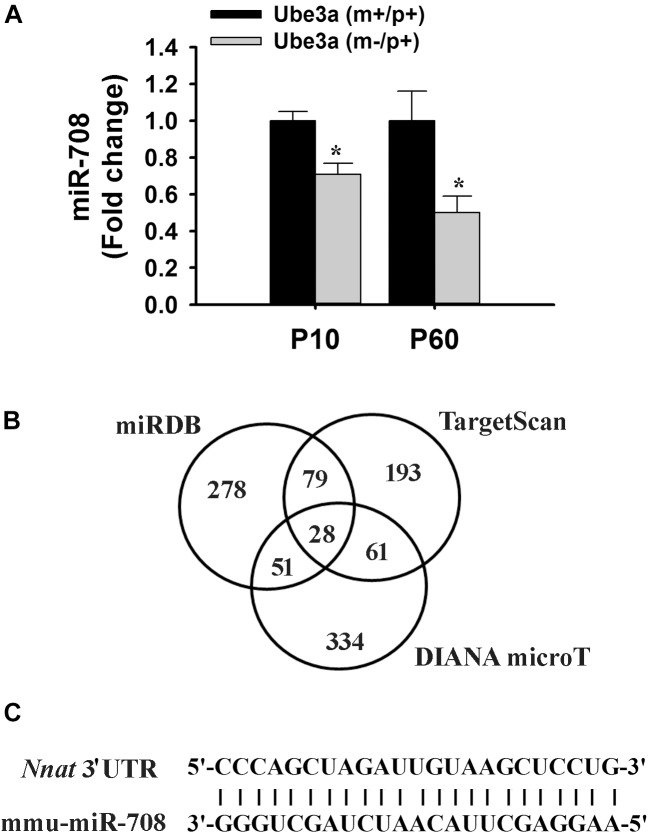
Down-regulation of miR-708 in AS mice brain and identification of Nnat as one of its predicted target. **(A)** Quantitative RT-PCR analysis of miR-708 from the total RNA extracted from the cortex sample of wild type and AS mice at postnatal day 10 and 60 (P10 and P60). Each sample was tested in triplicate and normalized against RNU6 (internal control). Values are mean ± SD with four animals/group at P10 and six animals/group at P60. ^∗^*P* < 0.001 as compared to respective wild type group. **(B)** Venn diagram depicting the number of identified target gene predicted by three different algorithms. **(C)** Sequence of miR-708 and the probable binding site at the 3′-UTR of *Nnat* that shows 100% resemblance.

In order to identify miRNAs that are regulated by Ube3a or deregulated in *Ube3a*-maternal deficient mice (AS model mice) brain, we performed miRNA array using total RNA extracted from cortical brain samples of 2 months old wild type and AS mice. Several deregulated miRNAs were identified in the cortical tissue of AS mice compared to wild type control (detailed list provided in Supplementary Information). A list of top up and down-regulated miRNAs were shown in Table 1. From the list of differentially regulated miRNA, we initially focused our study on miR-708 (one of the down-regulated miRNA), because of its strong association with bipolar disorder (Forstner et al., 2015; Fiorentino et al., 2016). Besides, its mis-regulation is not reported in any neurodevelopmental disorders. To further validate the decreased expression of miR-708 in AS mice brain, we analyzed the cortical sample (obtained from wild type and AS mice at P10 and P60) using quantitative real time PCR and observed a significant reduction in the expression of this miRNA in AS mice samples with respect to wild type controls (Figure 1A). The expression of miR-708 was reduced to about 30% at P10 and 50% at P60 in AS mice brain. Moreover, the relative expression of miR-708 in the cortical area of wild type mice was about two fold lower (at both P10 and P60) when compared with some of the brain-enriched miRNA like miR-34a or let-7a. We next aimed to identify the target of miR-708 using three target predicting algorithms (TargetScan, DIANA microT and miRDB). Among 28 possible targeted genes identified by all three algorithms (Figure 1B), we decided to study Nnat because of its developmentally regulated expression and possible link with brain development (Joseph et al., 1995; Joseph, 2014). Analysis of 3′-UTR sequences of *Nnat* showed one binding site that entirely matches with the sequence of miR-708 (Figure 1C). The sequence of miR-708 and its binding site in *Nnat* are also highly conserved across species.

**Table 1:** List of top up and down-regulated miRNA in the cortical sample of 2 months old AS mice.

S. no	Upregulated miRNA	Fold change	Down regulated miRNA	Fold change
1	mmu-miR-490	3.15	mmu-miR-539	2.69
2	mmu-miR-706	2.68	mmu-miR-466b	2.67
3	mmu-miR-214	2.47	mmu-miR-7b	2.62
4	mmu-miR-423	2.43	mmu-miR-377	2.57
5	mmu-miR-320	2.34	mmu-miR-669b	2.52
6	mmu-miR-665	2.32	mmu-miR-362	2.29
7	mmu-miR-370	2.23	mmu-miR-708	2.20
8	mmu-miR-700	2.20	mmu-miR-203	2.20
9	mmu-miR-760	2.19	mmu-miR-148b	2.12
10	mmu-miR-770	2.19	mmu-miR-1983	2.11

*Total RNA was isolated from the cortex sample (obtained from two mice in wild type and AS group) and then processed for microRNA array using Affymatrix miRNA 3 array and data were analyzed using GeneSpring GX12.5 software*.

### miR-708 Regulates the Expression of *Nnat*

**FIGURE 2:**
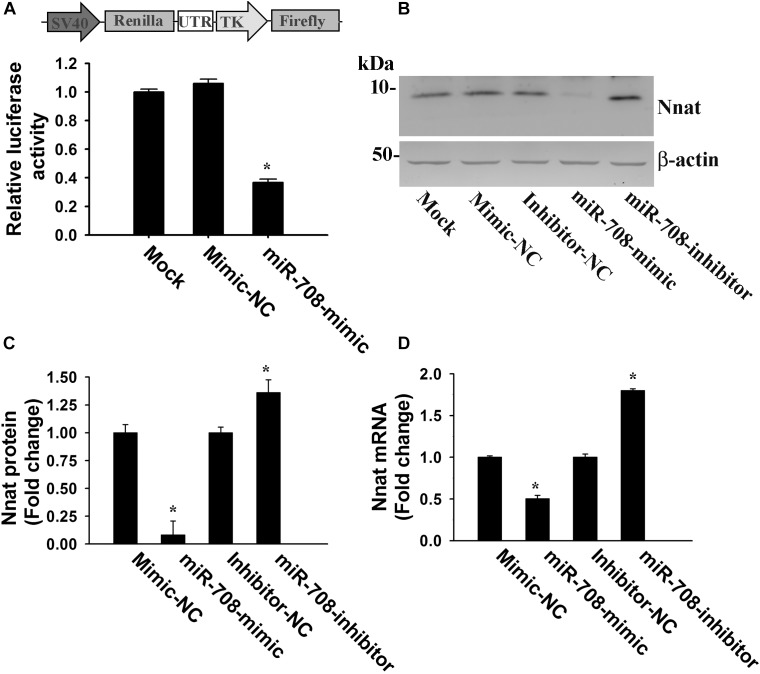
Regulation of Nnat expression by miR-708. **(A)** The 3′-UTR of *Nnat* was cloned into a dual luciferase reporter vector and then transfected into neuro 2a cells in the presence of miR-708 mimic or mimic negative control (NC). Twenty four hours after transfection, cells were processed for dual-luciferase assay. **(B–D)** Neuro 2a cells were transiently transfected with miR-708 mimic, inhibitor and their negative controls separately and 24 h later cells were collected and subjected to either immunoblot or quantitative PCR analysis of Nnat. **(B)** Representative immunoblot of Nnat along with β-actin. **(C)** Quantitative analysis of band intensity of Nnat shown in B using NIH Image analysis software. **(D)** Quantitative RT-PCR analysis of Nnat mRNA levels. Values are mean ± SD of three independent experiments. ^∗^*P* < 0.001 compared to respective control group.

To confirm whether miR-708 directly targets the 3′-UTR of *Nnat*, we cloned the 3′-UTR of *Nnat* into a dual-luciferase UTR reporter plasmid and co-transfected along with either miR-708 mimic or negative control into neuro 2a cells. Cells were collected at 24 h of post-transfection and processed for dual luciferase assay. As shown in Figure 2A, miR-708 mimic significantly inhibited the firefly luciferase activity as compared to control. Transfection of miR-708 mimic also resulted in significant reduction in the level of Nnat (Figure 2B,C). Similarly, transfection of miR-708 inhibitor caused increased expression of Nnat (Figure 2B,C). The mRNA level of Nnat was also negatively or positively regulated by miR-708 mimic or its inhibitor respectively (Figure 2D).

### Nnat Expression Is Increased in AS Mice Brain

**FIGURE 3:**
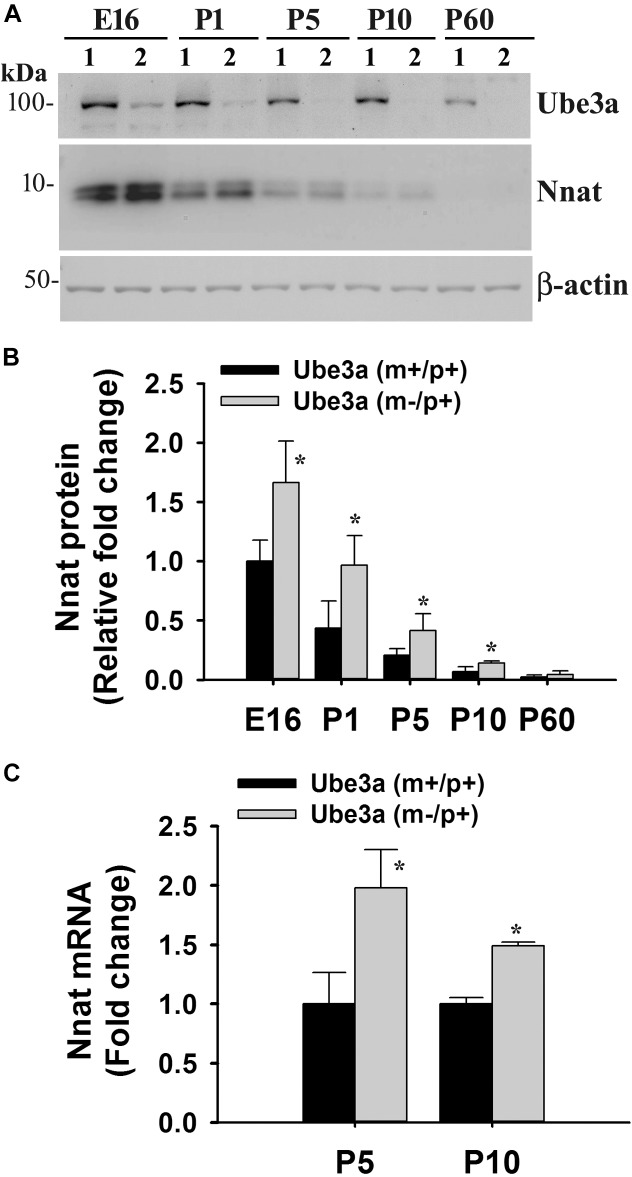
Increased expression of Nnat in AS mice brain. Cortical region of the brain from wild type and AS mice at their different ages (shown in figure) were isolated and subjected to either immunoblot or quantitative RT-PCR analysis of Nnat. **(A)** Representative immunoblots of Nnat, Ube3a and β-actin. Lane numbers 1 and 2 represents samples from wild type and AS mice respectively. **(B)** Quantitative analysis of Nnat level shown in **(A)**. **(C)** Quantitative RT-PCR analysis of Nnat mRNA. Data represent mean ± SD with three animals in each age group of wild type and AS mice. ^∗^*P* < 0.01 compared to respective control group. E, embryonic; P, postnatal.

**FIGURE 4:**
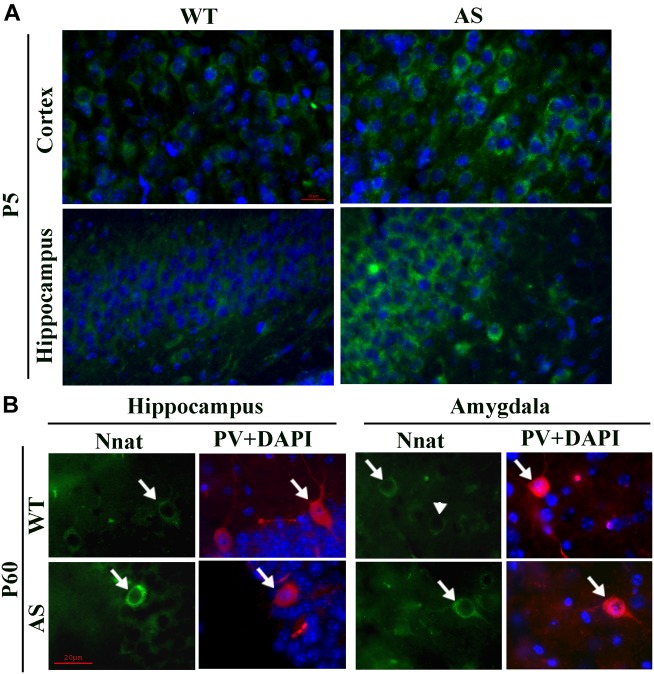
Comparative analysis of immunofluorescence staining of Nnat in various brain regions between wild type and AS mice at postnatal day P5 and P60. **(A)** Typical immunofluorescence staining of Nnat in the cortical and hippocampal region of wild type and AS mice. **(B)** Double immunofluorescence staining of Nnat and PV in the brain section obtained from 60 days old (P60) wild type and AS mice. Note the selective localization of Nnat in PV neurons (indicated by arrow). Arrowhead points the Nnat stained neuron that is not localized with PV neuron. Brain sections obtained from both wild type and AS mice were kept on the same slide and processed for immunostaining. Scale: 20 μm.

To explore the impact of decreased expression of miR-708 on its target Nnat in AS mice brain, we analyzed the expression of Nnat and compared with age-matched wild type control group. Cortical samples were collected from wild type and AS mice brain at different ages and then processed for immunoblot analysis using Nnat and Ube3a antibodies. In wild type mice, Nnat was found to be highly expressed in embryonic day 16 (E16) and then gradually decreased and was undetectable at postnatal day 60 (P60). However, Nnat level was significantly higher in AS mice samples from E16 to P10 when compared with respective age-matched wild type controls (Figure 3A,B). Ube3a expression was detectable at low level at E16 in AS mice, which gradually decreased and was undetectable from P5. AS mice cortical samples also exhibited a significantly increased level of Nnat mRNA in comparison with control at P5 and P10 (Figure 3C). We next carried out immunofluorescence staining of Nnat to further analyze its expression in different brain regions and subcellular distribution in wild type and AS mice brain at P5 and P60. At P5, Nnat was widely expressed throughout the brain and in AS mice its level was comparatively increased in most brain regions with respect to wild type mice (Figure 4A and Supplementary Figure S1 for smaller magnification images). Nnat was predominantly localized in the cell soma and to a lesser level in neuronal processes. Interestingly, at P60, Nnat expression was restricted to hippocampus, amygdala, hypothalamus, certain layer of cortex and a specific cell type was seen to be highly expressed. Earlier, we have shown that Nnat is expressed in parvalbumin (PV) types of GABAergic neurons in adult human brain (Sharma et al., 2011). We have confirmed that in normal adult mice brain, Nnat was not only expressed in PV+ve neurons but also in other types of neurons whose identities are still unknown (Supplementary Figure S2). Interestingly, level of Nnat in PV neurons was comparatively higher in AS mice with regard to wild type controls (Figure 4B).

### miR-708 Regulated Nnat Influence Intracellular Ca^2+^ Homeostasis

**FIGURE 5:**
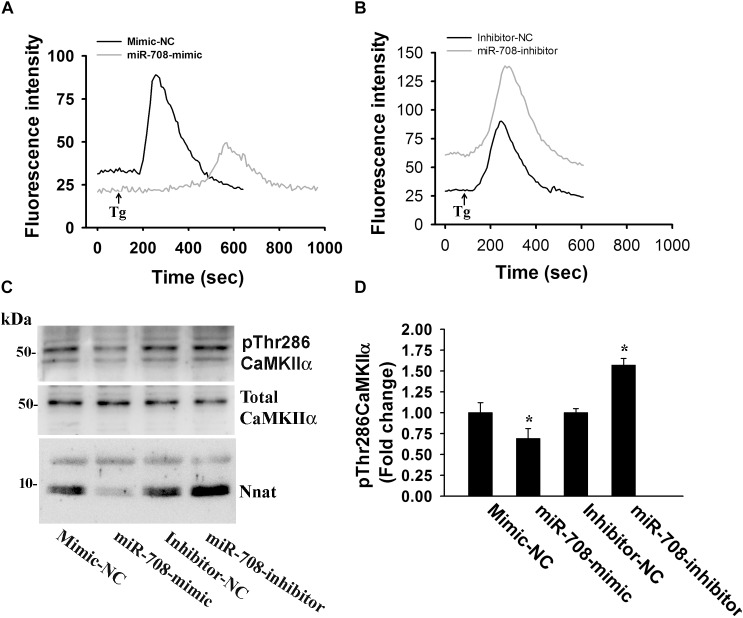
miR-708 regulates intracellular Ca^2+^ level and altered the phosphorylation of CAMKIIα at Thr286. Representative Ca^2+^ traces of neuro 2a cells transiently transfected with either miR-708 mimic **(A)** or miR-708 inhibitor **(B)** in the absence or presence of thapsigargin (Tg). Twenty four hours of post-transfection, cells were subjected to Ca^2+^ imaging using Fluo-4 AM dye. Thapsigargin (100 μM) was added after 100 sec of baseline recording. Approximately 50–60 cells were randomly selected in each experimental group for measuring fluorescence intensity and the experiment was repeated twice. **(C)** Immunoblot analysis of total and Thr286 phosphorylated form of CaMKIIα in miR-708 mimic or its inhibitor transfected neuro 2a cell lysates. **(D)** Quantitative evaluation of the band intensity of Thr286 phosphorylated form of CaMKIIα in the experiment shown in **(C)**. Data was normalized against total CaMKIIα. Values are mean ± SD of three independent experiments. ^∗^*P* < 0.01 compared to respective control group.

**FIGURE 6:**
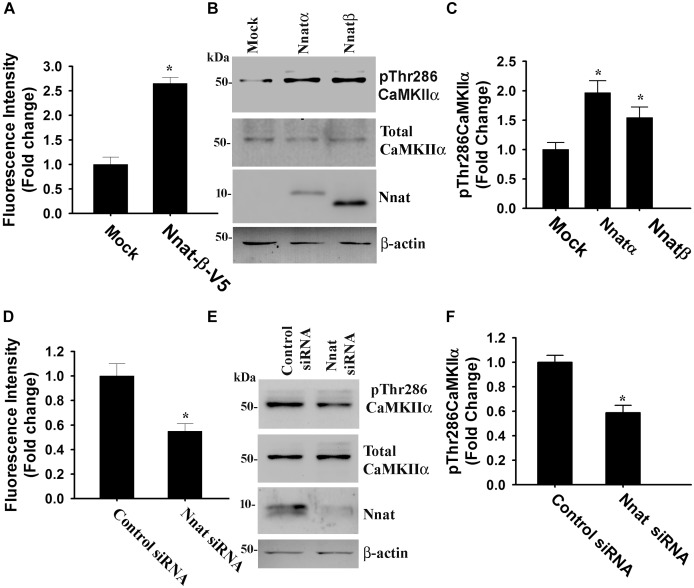
Nnat modulates intracellular Ca^2+^ homeostasis and the phosphorylation of CAMKIIα at Thr286. HT22 hippocampal cells were transiently transfected with Nnat expression plasmids (Nnatα and Nnatβ) for 24 h and then cells were either subjected to Ca^2+^ imaging experiment **(A)** or processed for immunoblotting using antibodies against total and Thr286 phosphorylated form of CaMKIIα **(B)** followed by quantitation of Thr286 phosphorylated form of CaMKIIα **(C)**. Over-expressed Nnatα and Nnatβ were detected by V5 antibody. Neuro 2a cells were transfected with Nnat siRNA and 24 h post-transfection, cells were either subjected to Ca^2+^ imaging **(D)** or processed for immunoblotting using antibodies against total and Thr286 phosphorylated form of CaMKIIα **(E)** followed by quantitation of Thr286 phosphorylated form of CaMKIIα **(F)**. About 50–60 randomly selected cells were assessed for measuring fluorescence intensity in Ca^2+^ imaging experiment and the experiment was repeated twice. Immunoblotting experiment was repeated thrice. Values are mean ± SD. ^∗^*P* < 0.01 compared to respective control group.

Nnat is localized in the ER and shown to antagonize the function of SERCA. This protein is also implicated in neural induction of embryonic stem cells possibly by increasing the intracellular Ca^2+^ level (Lin et al., 2010). Therefore, we presumed that miR-708-mediated regulation of Nnat might alter intracellular Ca^2+^ homeostasis and the downstream signaling pathways. To check our hypothesis, we transiently expressed miR-708 mimic or inhibitor into neuro 2a cells for 24 h and then measured the intracellular Ca^2+^ in the absence or presence of thapsigargin (an inhibitor of SERCA). In the control cell, thapsigargin treatment caused a rapid increase in cytosolic Ca^2+^ that quickly returned back to basal level. However, in the miR-708 transfected cell, basal cytosolic Ca^2+^ level was comparatively low and thapsigargin treatment resulted in delayed and a smaller amount of increased Ca^2+^ when compared to control (Figure 5A). Inhibitor of miR-708 produced completely opposite effect (Figure 5B). Since, intracellular Ca^2+^ activates multiple signaling pathways including CaMKs and extracellular signal-regulated kinases (ERKs), we further analyzed the phosphorylation of CaMKIIα at Thr286 because increased phosphorylation at this site of CaMKIIα along with impaired LTP was observed in AS mice brain (Weeber et al., 2003). We found that miR-708 mimic significantly decreased while its inhibitor increased the phosphorylation of CaMKIIα at Thr286 in neuro2a cells (Figure 5C,D). Next we tested the direct effect of Nnat in regulating intracellular Ca^2+^ levels either by knocking down or overexpressing Nnat. We observed that ectopic expression of Nnat α and β in HT22 hippocampal cell line (that does not express Nnat), caused significant increase in basal intracellular Ca^2+^ and increased phosphorylation of CaMKIIα at Thr286 (Figure 6A–C). Partial knockdown of Nnat in neuro 2a cells give rise to opposite effect (Figure 6D–F).

### Primary Cortical Neurons From AS Mice Show Increased Expression of Nnat and Augmented Ca^2+^ Signaling

**FIGURE 7:**
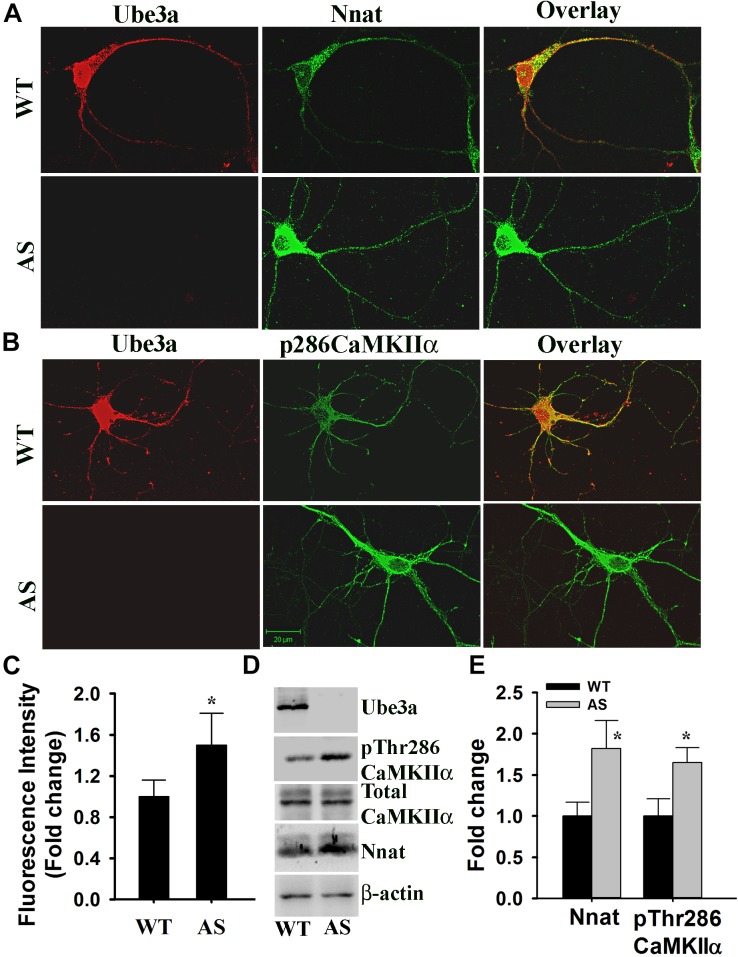
Primary cultured cortical neurons (E16 + 14DIV) prepared from AS mice embryos demonstrate increased expression of Nnat along with higher basal intracellular Ca^2+^ and increased phosphorylation of CaMKIIα at Thr286. **(A)** Double immunofluorescence staining of Nnat and Ube3a in the cortical neuron prepared from wild type and AS embryos. **(B)** Double immunofluorescence staining of pThr286 CaMKIIα and Ube3a showing increased phosphorylation of CaMKIIα at Thr286 in the cortical neuron cultured from AS mice embryo. At least 20 immunostained neurons from three different embryos in each group were assessed and most Ube3a-deficient neurons showed increased level of Nnat and pCaMKIIα at Thr286. **(C)** Comparison of intracellular basal Ca^2+^ level in the primary cortical neuron (E16 + 14 DIV) obtained from wild type and AS embryos using Fluo-4 AM dye. About 50–60 cells prepared from three different embryos in each group were used for evaluation. **(D,E)** Immunoblot analysis of Ube3a, Nnat and total and Thr286 phosphorylated form of CaMKIIα in the lysate prepared from primary cultured cortical neurons. Nnat level was normalized against β-actin while pThr286CaMKIIα was normalized against total CaMKIIα. Values are mean ± SD of three independent experiments. ^∗^*P* < 0.05 compared to respective wild type control group.

Angelman syndrome mice exhibit increased phosphorylation of CaMKIIα at Thr286 site in their hippocampus and other brain regions. Since Nnat regulated intracellular Ca^2+^ homeostasis and its level was increased in AS mice brain compared to wild type control, we further explored altered Ca^2+^ homeostasis and downstream signaling particularly phosphorylation of CaMKIIα at Thr286 site in primary cultured cortical neurons prepared from wild type and AS mice brain. First we analyzed the level of Nnat along with Ube3a through double immunofluorescence staining in 14 DIV cortical neurons obtained from wild type and AS mice. The expression of Nnat was very high in the cortical neuron prepared from AS mice compared to the wild type control (Figure 7A). Nnat was localized not only in the cell body but also in the neuronal processes with punctate staining. Nnat was not co-localized with Ube3a. We furthermore observed increased phosphorylation of CaMKIIα at Thr286 in the primary cortical neuron of AS mice with regard to controls (Figure 7B). Cortical neurons from AS mice also demonstrated elevated (about 1.6-fold) basal intracellular Ca^2+^ when compared to cortical neurons from wild type mice (Figure 7C). Immunoblot analysis further confirmed the increased level of Nnat and Thr286 phosphorylated form of CaMKIIα (Figure 7D,E). These results demonstrate aberrant Ca^2+^ signaling in the primary cortical neuron of AS mice, which could be due to increased expression of Nnat.

## Discussion

In the present investigation, we report down-regulation of miR-708 in AS mice brain, which could lead to aberrant increase in intracellular Ca^2+^ level by targeting Nnat, a regulator of SERCA of the ER. Our results also indicate a critical role of Ube3a in regulating intracellular Ca^2+^ homeostasis and the downstream signaling cascades. It is becoming increasingly evident that miRNAs have profound impact in regulating cognitive function and are involved in the pathogenesis of numerous neuropsychiatric and neurodevelopmental disorders (Xu et al., 2010; Beveridge and Cairns, 2012; Mellios and Sur, 2012; Hicks and Middleton, 2016; Wu et al., 2016). A large number of deregulated miRNAs are reported in various neurodevelopmental disorders like autism, Rett syndrome, Down syndrome, Fragile X-syndrome, etc. and many of those are essentially linked with synaptic maturation and plasticity (Li et al., 2008; Urdinguio et al., 2010; Mellios and Sur, 2012; Xu et al., 2013; Mellios et al., 2014; Hicks and Middleton, 2016; Karaca et al., 2017; Thomas et al., 2017). Although the miR-708 deregulation is commonly observed in various cancer tissues (Saini et al., 2012; Ryu et al., 2013), its altered expression so far not been reported in any other neurodevelopmental disorders. However, miR-708 was found to be strongly associated with bipolar disorder suggesting a common target of miR-708 might be associated with both AS and bipolar disorder (Forstner et al., 2015; Fiorentino et al., 2016). Interestingly, miR-708 is encoded within the intronic area of *Odz4* gene, which has been shown to regulate neuronal development and connectivity and its mutation is associated with bipolar disorder (Tucker and Chiquet-Ehrismann, 2006; Heinrich et al., 2013; Ikeda et al., 2017). Therefore, down-regulation of *Odz4* gene also could contribute to AS pathogenesis. The mechanistic basis of Ube3a-mediated regulation of miR-708 is not clear presently. The miR-708 has been shown to be regulated by polycomb group of transcriptional repressor complex as well as transcription factor CHOP (CCAAT enhancer binding protein homologous protein) (Behrman et al., 2011; Ryu et al., 2013). Interestingly, polycomb repressor complex is a target of Ube3a-mediated degradation (Zaaroor-Regev et al., 2010). Therefore, it is possible that the polycomb group of transcriptional repressor complex might be involved in regulating Ube3a-mediated regulation of miR-708.

Among several possible identified targets of miR-708, we focused our study on Nnat, because it is highly expressed during embryonic and early postnatal days and possibly involved in maintaining intracellular Ca^2+^ homeostasis by regulating SERCA pump (Lin et al., 2010). Nnat is also demonstrated to act as a novel intrinsic factor to promote neural induction in embryonic stem cells (Lin et al., 2010). Further, the miR-708 and its target site at 3′ UTR of Nnat are evolutionary conserved among mouse and human. We first experimentally validated Nnat as a target of miR-708 and our results were very similar with the recent observation made by others (Ryu et al., 2013). Subsequently, we observed increased expression of Nnat in the brain of AS mice during fetal and early postnatal days with respect to wild type control. We further dissected out the physiological function of Nnat and confirmed that it regulates intracellular Ca^2+^ homeostasis. Overexpression of Nnat or miR-708 inhibitor is able to significantly increase the basal as well as thapsigargin-stimulated intracellular Ca^2+^ level in neuronal cells. Furthermore, differentiated primary cortical neurons derived from AS mice also exhibited significantly higher Nnat along with increased intracellular basal Ca^2+^ level with respect to control. These results indicate that Ca^2+^ homeostasis is disrupted in the neuron of AS mice and aberrant Ca^2+^ signaling might contribute to disease pathogenesis. In fact, increased phosphorylation of CAMKIIα at constitutively active Thr286 site in Nnat-overexpressed hippocampal cell line as well as in differentiated primary cortical neurons from AS mice further support our observation. Increased phosphorylation of CAMKIIα at Thr286 was detected not only in the hippocampus but also in other regions of AS mice brain (Weeber et al., 2003; Mulherkar and Jana, 2010). Increased level of Nnat in AS mice brain might contribute to increased phosphorylation of CAMKIIα at Thr286 during early postnatal days (may be up to P10) and after that Nnat expression was very low and restricted to only limbic structures. Therefore, other factors like reduced protein phosphatase activity (PP1/PP2A) could be the best possible explanation for the increased phosphorylation of CAMKIIα at Thr286 in adult AS mice brain, which has been demonstrated earlier (Weeber et al., 2003). The increased phosphorylation of CAMKIIα at constitutively active site Thr286 also accompanied with its auto inhibitory phosphorylation at Thr305 resulting in overall inhibition of CAMKIIα activity in AS mice brain (Weeber et al., 2003). Interestingly, reduction in auto-inhibitory phosphorylation rescue neurological deficits in AS mice (van Woerden et al., 2007). Thus our findings of Nnat-induced Ca^2+^ signaling might be implicated in aberrant phosphorylation of CAMKIIα in AS mice brain.

Another interesting aspect of our study is the increased expression of Nnat in PV types of GABAergic interneurons of AS mice brain. Although, overall Nnat expression was dramatically reduced in normal adult brain, it is found to be specifically expressed in various limbic regions like hippocampus, amygdala, and hypothalamus (Vrang et al., 2010). Our observation of selective expression of Nnat in the PV type of GABAergic neuron suggests that Nnat might be involved in regulating Ca^2+^ homeostasis and GABA release from PV neurons. The increased level of Nnat in the PV neuron of AS mice brain could lead to aberrant GABAergic transmission or even dysfunction of these neurons. Elevated GABA was detected in the plasma of AS patients and there was also evidence of increased miniature inhibitory post-synaptic current (mIPSPs) frequency in AS mice hippocampus (Ebert et al., 1997; Kaphzan et al., 2012). Furthermore, blockade of inhibitory transmission by Erb (epidermal growth factor receptor predominantly localized in GABAergic neurons) inhibitor rescued hippocampal LTP impairment and contextual fear memory deficits in AS mice (Kaphzan et al., 2012). AS mice also exhibit reduced number of PV types of GABAergic neurons in their stress-sensitive brain regions including hippocampus and amygdala (Godavarthi et al., 2014). These finding indicates that there could be dysfunction of at least PV types of GABAergic neurons in AS brain that eventually might lead to imbalance of excitatory/inhibitory circuitry, underlying cause of many behavioral deficits including increased susceptibility to seizure.

## Conclusion

Our study identified miR-708 as one of the novel down-regulated miRNA in AS mice brain. The deregulated miR-708 and its target Nnat could lead to aberrant Ca^+^ signaling, which might underlie AS pathogenesis.

## Author Contributions

NJ conceived the study and wrote the manuscript. NV, VK, BS, SK, and AS performed the experiments. NV and VK analyzed the data. All authors reviewed the manuscript.

## Conflict of Interest Statement

The authors declare that the research was conducted in the absence of any commercial or financial relationships that could be construed as a potential conflict of interest.

## References

[B1] Albrecht U. Sutcliffe J. S. Cattanach B. M. Beechey C. V. Armstrong D. Eichele G. (1997). Imprinted expression of the murine Angelman syndrome gene, Ube3a, in hippocampal and Purkinje neurons. *Nat. Genet.* 17 75–78. 10.1038/ng0997-75 9288101

[B2] Alvarez-Garcia I. Miska E. A. (2005). MicroRNA functions in animal development and human disease. *Development* 132 4653–4662. 10.1242/dev.02073 16224045

[B3] Baek D. Villen J. Shin C. Camargo F. D. Gygi S. P. Bartel D. P. (2008). The impact of microRNAs on protein output. *Nature* 455 64–71. 10.1038/nature07242 18668037 PMC2745094

[B4] Bartel D. P. (2009). MicroRNAs: target recognition and regulatory functions. *Cell* 136 215–233. 10.1016/j.cell.2009.01.002 19167326 PMC3794896

[B5] Behrman S. Acosta-Alvear D. Walter P. (2011). A CHOP-regulated microRNA controls rhodopsin expression. *J. Cell Biol.* 192 919–927. 10.1083/jcb.201010055 21402790 PMC3063143

[B6] Beveridge N. J. Cairns M. J. (2012). MicroRNA dysregulation in schizophrenia. *Neurobiol. Dis.* 46 263–271. 10.1016/j.nbd.2011.12.029 22207190

[B7] Coolen M. Bally-Cuif L. (2009). MicroRNAs in brain development and physiology. *Curr. Opin. Neurobiol.* 19 461–470. 10.1016/j.conb.2009.09.006 19846291

[B8] Dou D. Joseph R. (1996). Cloning of human neuronatin gene and its localization to chromosome-20q 11.2*-*12: the deduced protein is a novel “proteolipid”. *Brain Res.* 723 8–22. 10.1016/0006-8993(96)00167-9 8813377

[B9] Ebert M. H. Schmidt D. E. Thompson T. Butler M. G. (1997). Elevated plasma gamma-aminobutyric acid (GABA) levels in individuals with either Prader-Willi syndrome or Angelman syndrome. *J. Neuropsychiatry Clin. Neurosci.* 9 75–80. 10.1176/jnp.9.1.75 9017532 PMC5972534

[B10] Fang P. Lev-Lehman E. Tsai T. F. Matsuura T. Benton C. S. Sutcliffe J. S. (1999). The spectrum of mutations in UBE3A causing Angelman syndrome. *Hum. Mol. Genet* 8 129–135. 10.1093/hmg/8.1.129 9887341

[B11] Fineberg S. K. Kosik K. S. Davidson B. L. (2009). MicroRNAs potentiate neural development. *Neuron* 64 303–309. 10.1016/j.neuron.2009.10.020 19914179

[B12] Fiorentino A. O’Brien N. L. Sharp S. I. Curtis D. Bass N. J. McQuillin A. (2016). Genetic variation in the miR-708 gene and its binding targets in bipolar disorder. *Bipolar Disord.* 18 650–656. 10.1111/bdi.12448 27864917 PMC5244671

[B13] Forstner A. J. Hofmann A. Maaser A. Sumer S. Khudayberdiev S. Muhleisen T. W. (2015). Genome-wide analysis implicates microRNAs and their target genes in the development of bipolar disorder. *Transl. Psychiatry* 5:e678. 10.1038/tp.2015.159 26556287 PMC5068755

[B14] Glessner J. T. Wang K. Cai G. Korvatska O. Kim C. E. Wood S. (2009). Autism genome-wide copy number variation reveals ubiquitin and neuronal genes. *Nature* 459 569–573. 10.1038/nature07953 19404257 PMC2925224

[B15] Godavarthi S. K. Dey P. Maheshwari M. Jana N. R. (2012). Defective glucocorticoid hormone receptor signaling leads to increased stress and anxiety in a mouse model of Angelman syndrome. *Hum. Mol. Genet.* 21 1824–1834. 10.1093/hmg/ddr614 22215440

[B16] Godavarthi S. K. Sharma A. Jana N. R. (2014). Reversal of reduced parvalbumin neurons in hippocampus and amygdala of Angelman syndrome model mice by chronic treatment of fluoxetine. *J. Neurochem.* 130 444–454. 10.1111/jnc.12726 24678582

[B17] Greer P. L. Hanayama R. Bloodgood B. L. Mardinly A. R. Lipton D. M. Flavell S. W. (2010). The Angelman Syndrome protein Ube3A regulates synapse development by ubiquitinating arc. *Cell* 140 704–716. 10.1016/j.cell.2010.01.026 20211139 PMC2843143

[B18] Heck D. H. Zhao Y. Roy S. LeDoux M. S. Reiter L. T. (2008). Analysis of cerebellar function in Ube3a-deficient mice reveals novel genotype-specific behaviors. *Hum. Mol. Genet.* 17 2181–2189. 10.1093/hmg/ddn117 18413322 PMC2902285

[B19] Heinrich A. Lourdusamy A. Tzschoppe J. Vollstadt-Klein S. Buhler M. Steiner S. (2013). The risk variant in ODZ4 for bipolar disorder impacts on amygdala activation during reward processing. *Bipolar Disord.* 15 440–445. 10.1111/bdi.12068 23611537

[B20] Hicks S. D. Middleton F. A. (2016). A comparative review of microRNA expression patterns in autism spectrum disorder. *Front. Psychiatry* 7:176. 10.3389/fpsyt.2016.00176 27867363 PMC5095455

[B21] Huibregtse J. M. Scheffner M. Beaudenon S. Howley P. M. (1995). A family of proteins structurally and functionally related to the E6-AP ubiquitin-protein ligase. *Proc. Natl. Acad. Sci. U.S.A.* 92 2563–2567. 10.1073/pnas.92.7.2563 7708685 PMC42258

[B22] Ikeda M. Takahashi A. Kamatani Y. Okahisa Y. Kunugi H. Mori N. (2017). A genome-wide association study identifies two novel susceptibility loci and trans population polygenicity associated with bipolar disorder. *Mol. Psychiatry* 23 639–647. 10.1038/mp.2016.259 28115744 PMC5822448

[B23] Im H. I. Kenny P. J. (2012). MicroRNAs in neuronal function and dysfunction. *Trends Neurosci.* 35 325–334. 10.1016/j.tins.2012.01.004 22436491 PMC3565236

[B24] Inui M. Martello G. Piccolo S. (2010). MicroRNA control of signal transduction. *Nat. Rev. Mol. Cell Biol.* 11 252–263. 10.1038/nrm2868 20216554

[B25] Jiang Y. H. Armstrong D. Albrecht U. Atkins C. M. Noebels J. L. Eichele G. (1998). Mutation of the Angelman ubiquitin ligase in mice causes increased cytoplasmic p53 and deficits of contextual learning and long-term potentiation. *Neuron* 21 799–811. 10.1016/S0896-6273(00)80596-6 9808466

[B26] Joseph R. Dou D. Tsang W. (1995). Neuronatin mRNA: alternatively spliced forms of a novel brain-specific mammalian developmental gene. *Brain Res.* 690 92–98. 10.1016/0006-8993(95)00621-V 7496812

[B27] Joseph R. M. (2014). Neuronatin gene: imprinted and misfolded: studies in Lafora disease, diabetes and cancer may implicate NNAT-aggregates as a common downstream participant in neuronal loss. *Genomics* 103 183–188. 10.1016/j.ygeno.2013.12.001 24345642

[B28] Kaphzan H. Hernandez P. Jung J. I. Cowansage K. K. Deinhardt K. Chao M. V. (2012). Reversal of impaired hippocampal long-term potentiation and contextual fear memory deficits in Angelman syndrome model mice by ErbB inhibitors. *Biol. Psychiatry* 72 182–190. 10.1016/j.biopsych.2012.01.021 22381732 PMC3368039

[B29] Karaca E. Aykut A. Erturk B. Durmaz B. Guler A. Buke B. (2017). Diagnostic role of MicroRNA expression profile in the prenatal amniotic fluid samples of pregnant women with down syndrome. *Balkan Med. J.* 35 163–166. 10.4274/balkanmedj.2017.0511 29219113 PMC5863254

[B30] Kishino T. Lalande M. Wagstaff J. (1997). UBE3A/E6-AP mutations cause Angelman syndrome. *Nat. Genet.* 15 70–73. 10.1038/ng0197-70 8988171

[B31] Li Y. Lin L. Jin P. (2008). The microRNA pathway and fragile X mental retardation protein. *Biochim. Biophys. Acta* 1779 702–705. 10.1016/j.bbagrm.2008.07.003 18687414 PMC2607293

[B32] Lin H. H. Bell E. Uwanogho D. Perfect L. W. Noristani H. Bates T. J. (2010). Neuronatin promotes neural lineage in ESCs via Ca(2+) signaling. *Stem Cells* 28 1950–1960. 10.1002/stem.530 20872847 PMC3003906

[B33] Liu T. Wan R. P. Tang L. J. Liu S. J. Li H. J. Zhao Q. H. (2015). A microRNA profile in fmr1 knockout mice reveals MicroRNA expression alterations with possible roles in fragile x syndrome. *Mol. Neurobiol.* 51 1053–1063. 10.1007/s12035-014-8770-1 24906954

[B34] Matsuura T. Sutcliffe J. S. Fang P. Galjaard R. J. Jiang Y. H. Benton C. S. (1997). De novo truncating mutations in E6-AP ubiquitin-protein ligase gene (UBE3A) in Angelman syndrome. *Nat. Genet.* 15 74–77. 10.1038/ng0197-74 8988172

[B35] Mellios N. Sur M. (2012). The emerging role of microRNAs in schizophrenia and autism spectrum disorders. *Front. Psychiatry* 3:39. 10.3389/fpsyt.2012.00039 22539927 PMC3336189

[B36] Mellios N. Woodson J. Garcia R. I. Crawford B. Sharma J. Sheridan S. D. (2014). beta2-Adrenergic receptor agonist ameliorates phenotypes and corrects microRNA-mediated IGF1 deficits in a mouse model of Rett syndrome. *Proc. Natl. Acad. Sci. U.S.A.* 111 9947–9952. 10.1073/pnas.1309426111 24958851 PMC4103343

[B37] Mulherkar S. A. Jana N. R. (2010). Loss of dopaminergic neurons and resulting behavioural deficits in mouse model of Angelman syndrome. *Neurobiol. Dis.* 40 586–592. 10.1016/j.nbd.2010.08.002 20696245

[B38] Mundalil Vasu M. Anitha A. Thanseem I. Suzuki K. Yamada K. Takahashi T. (2014). Serum microRNA profiles in children with autism. *Mol. Autism* 5:40. 10.1186/2040-2392-5-40 25126405 PMC4132421

[B39] Ramamoorthy S. Nawaz Z. (2008). E6-associated protein (E6-AP) is a dual function coactivator of steroid hormone receptors. *Nucl. Recept. Signal.* 6:e006. 10.1621/nrs.06006 18432313 PMC2329825

[B40] Ryu S. McDonnell K. Choi H. Gao D. Hahn M. Joshi N. (2013). Suppression of miRNA-708 by polycomb group promotes metastases by calcium-induced cell migration. *Cancer Cell* 23 63–76. 10.1016/j.ccr.2012.11.019 23328481

[B41] Saini S. Majid S. Shahryari V. Arora S. Yamamura S. Chang I. (2012). miRNA-708 control of CD44(+) prostate cancer-initiating cells. *Cancer Res.* 72 3618–3630. 10.1158/0008-5472.CAN-12-0540 22552290

[B42] Sato M. Stryker M. P. (2010). Genomic imprinting of experience-dependent cortical plasticity by the ubiquitin ligase gene Ube3a. *Proc. Natl. Acad. Sci. U.S.A.* 107 5611–5616. 10.1073/pnas.1001281107 20212164 PMC2851788

[B43] Sharma J. Mukherjee D. Rao S. N. Iyengar S. Shankar S. K. Satishchandra P. (2013). Neuronatin-mediated aberrant calcium signaling and endoplasmic reticulum stress underlie neuropathology in Lafora disease. *J. Biol. Chem.* 288 9482–9490. 10.1074/jbc.M112.416180 23408434 PMC3611017

[B44] Sharma J. Rao S. N. Shankar S. K. Satishchandra P. Jana N. R. (2011). Lafora disease ubiquitin ligase malin promotes proteasomal degradation of neuronatin and regulates glycogen synthesis. *Neurobiol. Dis.* 44 133–141. 10.1016/j.nbd.2011.06.013 21742036

[B45] Shi S. Q. Bichell T. J. Ihrie R. A. Johnson C. H. (2015). Ube3a imprinting impairs circadian robustness in Angelman syndrome models. *Curr. Biol.* 25 537–545. 10.1016/j.cub.2014.12.047 25660546 PMC4348236

[B46] Sun J. Zhu G. Liu Y. Standley S. Ji A. Tunuguntla R. (2010). UBE3A regulates synaptic plasticity and learning and memory by controlling SK2 channel endocytosis. *Cell Rep.* 12 449–461. 10.1016/j.celrep.2015.06.023 26166566 PMC4520703

[B47] Thomas K. T. Anderson B. R. Shah N. Zimmer S. E. Hawkins D. Valdez A. N. (2017). Inhibition of the schizophrenia-associated microRNA miR-137 disrupts Nrg1alpha neurodevelopmental signal transduction. *Cell Rep.* 20 1–12. 10.1016/j.celrep.2017.06.038 28683304 PMC5745041

[B48] Tucker R. P. Chiquet-Ehrismann R. (2006). Teneurins: a conserved family of transmembrane proteins involved in intercellular signaling during development. *Dev. Biol.* 290 237–245. 10.1016/j.ydbio.2005.11.038 16406038

[B49] Urdinguio R. G. Fernandez A. F. Lopez-Nieva P. Rossi S. Huertas D. Kulis M. (2010). Disrupted microRNA expression caused by Mecp2 loss in a mouse model of Rett syndrome. *Epigenetics* 5 656–663. 10.4161/epi.5.7.13055 20716963 PMC3052849

[B50] van Woerden G. M. Harris K. D. Hojjati M. R. Gustin R. M. Qiu S. de Avila Freire R. (2007). Rescue of neurological deficits in a mouse model for Angelman syndrome by reduction of alphaCaMKII inhibitory phosphorylation. *Nat. Neurosci.* 10 280–282. 10.1038/nn1845 17259980

[B51] Vrang N. Meyre D. Froguel P. Jelsing J. Tang-Christensen M. Vatin V. (2010). The imprinted gene neuronatin is regulated by metabolic status and associated with obesity. *Obesity* 18 1289–1296. 10.1038/oby.2009.361 19851307 PMC2921166

[B52] Wallace M. L. Burette A. C. Weinberg R. J. Philpot B. D. (2012). Maternal loss of Ube3a produces an excitatory/inhibitory imbalance through neuron type-specific synaptic defects. *Neuron* 74 793–800. 10.1016/j.neuron.2012.03.036 22681684 PMC3372864

[B53] Weeber E. J. Jiang Y. H. Elgersma Y. Varga A. W. Carrasquillo Y. Brown S. E. (2003). Derangements of hippocampal calcium/calmodulin-dependent protein kinase II in a mouse model for Angelman mental retardation syndrome. *J. Neurosci.* 23 2634–2644. 10.1523/JNEUROSCI.23-07-02634.2003 12684449 PMC6742065

[B54] Williams C. A. Driscoll D. J. Dagli A. I. (2010). Clinical and genetic aspects of Angelman syndrome. *Genet. Med.* 12 385–395. 10.1097/GIM.0b013e3181def138 20445456

[B55] Wu Y. E. Parikshak N. N. Belgard T. G. Geschwind D. H. (2016). Genome-wide, integrative analysis implicates microRNA dysregulation in autism spectrum disorder. *Nat. Neurosci.* 19 1463–1476. 10.1038/nn.4373 27571009 PMC5841760

[B56] Xu B. Karayiorgou M. Gogos J. A. (2010). MicroRNAs in psychiatric and neurodevelopmental disorders. *Brain Res.* 1338 78–88. 10.1016/j.brainres.2010.03.109 20388499 PMC2883644

[B57] Xu X. Li C. Gao X. Xia K. Guo H. Li Y. (2018). Excessive UBE3A dosage impairs retinoic acid signaling and synaptic plasticity in autism spectrum disorders. *Cell Res.* 28 48–68. 10.1038/cr.2017.132 29076503 PMC5752837

[B58] Xu Y. Li W. Liu X. Ma H. Tu Z. Dai Y. (2013). Analysis of microRNA expression profile by small RNA sequencing in Down syndrome fetuses. *Int. J. Mol. Med.* 32 1115–1125. 10.3892/ijmm.2013.1499 24071828

[B59] Yamasaki K. Joh K. Ohta T. Masuzaki H. Ishimaru T. Mukai T. (2003). Neurons but not glial cells show reciprocal imprinting of sense and antisense transcripts of Ube3a. *Hum. Mol. Genet.* 12 837–847. 10.1093/hmg/ddg106 12668607

[B60] Yashiro K. Riday T. T. Condon K. H. Roberts A. C. Bernardo D. R. Prakash R. (2009). Ube3a is required for experience-dependent maturation of the neocortex. *Nat. Neurosci.* 12 777–783. 10.1038/nn.2327 19430469 PMC2741303

[B61] Yi J. J. Berrios J. Newbern J. M. Snider W. D. Philpot B. D. Hahn K. M. (2015). An autism-linked mutation disables phosphorylation control of UBE3A. *Cell* 162 795–807. 10.1016/j.cell.2015.06.045 26255772 PMC4537845

[B62] Zaaroor-Regev D. de Bie P. Scheffner M. Noy T. Shemer R. Heled M. (2010). Regulation of the polycomb protein ring1B by self-ubiquitination or by E6-AP may have implications to the pathogenesis of Angelman syndrome. *Proc. Natl. Acad. Sci. U.S.A.* 107 6788–6793. 10.1073/pnas.1003108107 20351251 PMC2872415

