# Supplementary material for: Down-Regulation of miRNA-708 Promotes Aberrant Calcium Signaling by Targeting Neuronatin in a Mouse Model of Angelman Syndrome
Source: Front Mol Neurosci. 2019 Feb 13;12:35. doi: 10.3389/fnmol.2019.00035 (PMC6381399; doi:10.3389/fnmol.2019.00035)
Supplement: Supplementary file 1 [file Data_Sheet_1.PDF]

Supplementary information

Down-regulation of miRNA-708 promotes aberrant calcium signalling by targeting neuronatin in a mouse model of Angelman syndrome

Naman Vatsa, Vipendra Kumar, Brijesh Kumar Singh, Shashi Shekhar Kumar, Ankit Sharma and Nihar Ranjan Jana

Fig.S1. Immunofluorescence staining of Nnat in cortical, hippocampal and hypothalamic region of wild type and AS mice at P10.

Fig.S2. Double immunofluorescence staining of Nnat and PV in the brain section obtained from 60 days old wild type mice. Note the selective localization of Nnat in PV neurons (indicated by arrow). Arrowhead points the Nnat stained neuron that is not localized with PV neuron. Scale, 20µm.

Fig.S1

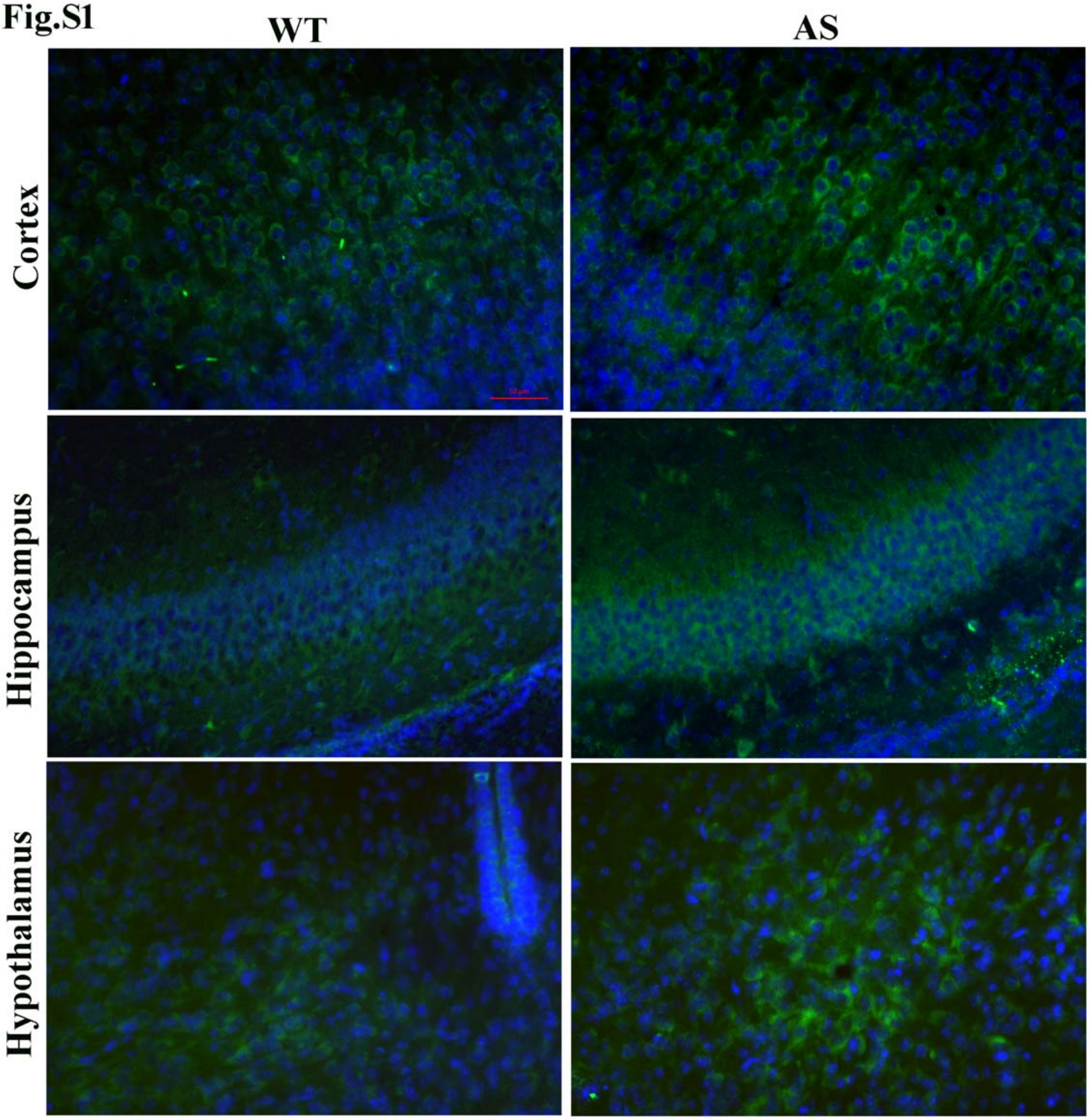

Fig.S2

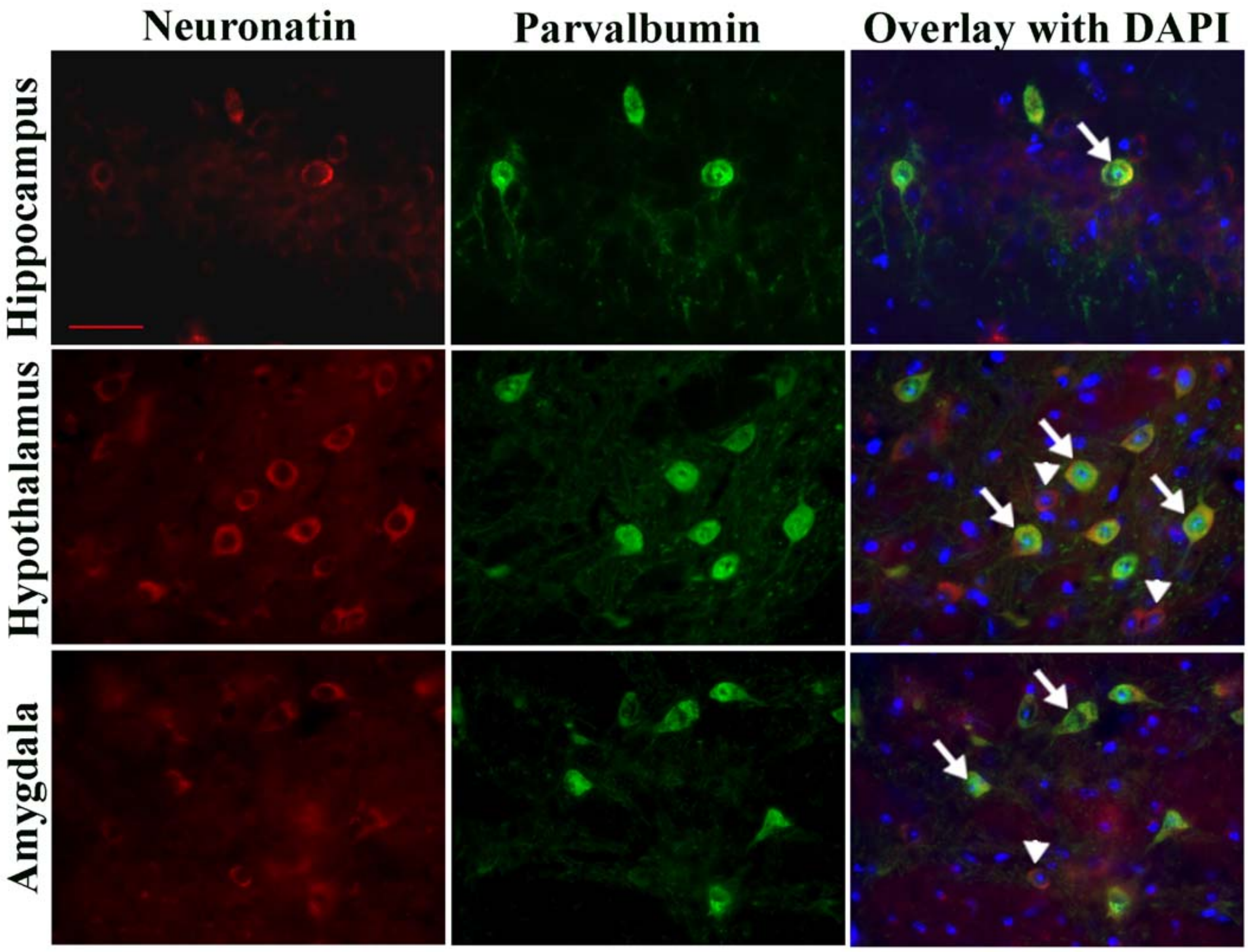
Neuronatin
Parvalbumin
Overlay with DAPI
Hippocampus
Hypothalamus
Amygdala
